# The Prognostic Role of Pitt Bacteremia Score in Patients With Nonbacteremic *Klebsiella pneumoniae* Infections

**DOI:** 10.1155/cjid/6780766

**Published:** 2025-07-15

**Authors:** Jia-Mei Chang, Kuo-Hsuan Lin, Chung-Hsu Lai, I-Ting Tsai, Yin-Chou Hsu

**Affiliations:** ^1^Department of Emergency Medicine, E-Da Hospital, I-Shou University, Kaohsiung 82445, Taiwan; ^2^School of Medicine, College of Medicine, I-Shou University, Kaohsiung, Taiwan; ^3^School of Chinese Medicine for Post Baccalaureate, I-Shou University, Kaohsiung, Taiwan; ^4^Division of Infectious Diseases, Department of Internal Medicine, E-Da Hospital, I-Shou University, Kaohsiung 82445, Taiwan; ^5^Department of Emergency Medicine, Yuan's General Hospital, Kaohsiung, Taiwan; ^6^Graduate Institute of Clinical Medicine, College of Medicine, Kaohsiung Medical University, Kaohsiung 807, Taiwan

**Keywords:** emergency department, *Klebsiella pneumoniae*, nonbacteremia, Pitt bacteremia score, prognosis

## Abstract

**Background:** *Klebsiella pneumoniae* infection causes various diseases and leads to significant morbidity and mortality. The Pitt bacteremia score (PBS) is a well-known prognostic predictor in patients with bacteremia. We aimed to investigate the prognostic role of the PBS in patients with nonbacteremic *K. pneumoniae* infections and compare its mortality discriminative ability with that of other risk scoring systems.

**Methods:** Data were retrospectively collected from emergency department patients in E-Da Hospital, Kaohsiung, Taiwan, within 2021. All adult patients (aged ≥ 20 years) during this period and diagnosed with *K. pneumoniae* infections were included. The baseline demographics, laboratory results, infection sources, and clinical outcomes of nonbacteremic patients were extracted, and the patients were further divided into low (< 4) and high (≥ 4) PBS groups for comparison.

**Results:** A total of 863 patients with *K. pneumoniae* infection were identified, and 639 nonbacteremic patients were enrolled. There were similar demographics between the bacteremic and nonbacteremic groups. Regarding clinical outcomes in nonbacteremic patients, the high PBS group had significantly higher risk of septic shock (77.9% vs. 4.8%, *p* < 0.01), intensive care unit admission (71.3% vs. 8.2%, *p* < 0.01), respiratory failure (71.3% vs. 2.4%, *p* < 0.01), and 30-day mortality (34.6% vs. 3.8%, *p* < 0.01). The area under the curve of the scoring systems regarding 30-day mortality prediction ability was as follows: sequential organ failure assessment score 0.89 (95% confidence interval [CI] = 0.86–0.91), PBS 0.86 (95% CI = 0.83–0.88), quick sequential organ failure assessment score 0.71 (95% CI = 0.67–0.74), and systemic inflammatory response syndrome 0.62 (95% CI = 0.58–0.66).

**Conclusion:** PBS correlated with adverse outcomes and good mortality prediction ability in patients with nonbacteremic *K. pneumoniae* infections.

## 1. Background

*Klebsiella pneumoniae* is a Gram-negative, encapsulated, nonmotile bacterium belonging to the family *Enterobacteriaceae* and a natural inhabitant of the mucosal surfaces of healthy humans, including the gastrointestinal tract and oropharynx [1, 2]. *K. pneumoniae* is recognized as an important causative pathogen of nosocomial infections and neonatal sepsis [3]. Although invasive *K. pneumoniae* infection is usually considered a result of opportunistic infections in immunocompromised patients, the emergence and spread of hypervirulent and antibiotic-resistant strains of *K. pneumoniae* have largely broadened the number of people susceptible to infections, including those with immunocompetent status [1]. The resistant strain of *K. pneumoniae* infection accounts for more than 200,000 annual deaths globally [4]. Notably, it has been estimated that the mortality rate due to *K. pneumoniae* nonbacteremic infection ranges from 10% to 19.5% [5, 6].

The Pitt bacteremia score (PBS), consisting of five variables (mental status, body temperature, hypotension or need for vasopressors, mechanical ventilation support, and cardiac arrest), was developed more than three decades ago to evaluate disease severity and predict mortality in patients with bacteremia [7]. It was originally created to describe and stratify the outcomes of patients with *Pseudomonas aeruginosa* bacteremia [7] and was validated for its prognostic significance in patients with other Gram-negative, Gram-positive, or even *Candida* pathogens [8–12]. Notably, a recent prospective, multicenter study of hospitalized patients with carbapenem-resistant *Enterobacteriaceae* infections demonstrated that PBS is also useful for outcome prediction in patients with nonbacteremic infections [13]. The straightforward calculation of PBS, based solely on physiological and clinical variables without needing to obtain laboratory results, allows for more rapid application and assessment in patients with infectious diseases, especially in those with critical illness [7, 13]. This study aimed to investigate the prognostic role of PBS in patients with nonbacteremic *K. pneumoniae* infections and to compare PBS with other commonly used risk stratification scoring systems in these patients.

## 2. Methods

### 2.1. Study Design and Population Enrollment

This single-center, retrospective observational cohort study was conducted in the emergency department (ED) of E-Da Hospital, Kaohsiung, Taiwan, between January 1, 2021, and December 31, 2021. The hospital has 1050 beds and 60 adult intensive care unit (ICU) beds. Approximately 65% of hospitalized patients were admitted from the ED source. All adult patients (aged ≥ 20 years) who visited the ED during this period and submitted to blood or other site-specific cultures that yield *K. pneumoniae* were enrolled. If patients had multiple ED visits within 30 days, only the first visit was analyzed [14, 15]. We excluded patients with nonhospitalization, colonization (did not meet the criteria of infection) [16, 17], and irrelevant diagnoses (only the cultured *K. pneumoniae* corresponding with the patient's principal diagnosis was collected). We also excluded patients with end-stage renal disease and chronic respiratory failure because these two comorbidities biased our analysis of adverse outcomes and calculation of PBS. This study followed the principles of the Declaration of Helsinki and the STROBE guidelines [18] and approved by the local institutional review board (EMRP-111-081). Because of the retrospective observational design of this study, the requirement for informed consent was waived by the ethics committee.

### 2.2. Data Collection

We collected all anonymized clinical information of each eligible patient, including baseline demographics, comorbidities, laboratory parameters, microbiological results, and outcomes, from the electronic medical record systems. Comorbidities were recognized by disease codes (International Classification of Diseases, 10th Revision), and laboratory results were obtained within 6 h of the ED visit for each patient. The enrolled patients were categorized into the bacteremic and nonbacteremic groups for further comparison. To avoid classification bias, only patients with primary bacteremia (i.e., bacteremia in the absence of an identified infection source) were classified into the bacteremic group. The sources of infection in the nonbacteremic group were stratified as follows: respiratory tract, urinary tract, intra-abdominal, skin/soft tissue, and others (minor infection sites). The resistant strain was considered if the cultured *K. pneumoniae* was resistant against the administered antimicrobial agent by in vitro susceptibility testing.

### 2.3. Definitions

Chronic kidney disease was defined as a baseline estimated glomerular filtration rate (eGFR) < 60 mL/min/1.73 m^2^, which was calculated using the simplified Modification of Diet in Renal Disease equation [19]. Systemic inflammatory response syndrome (SIRS) and quick sequential organ failure assessment (qSOFA) scores followed widely used criteria and were obtained by summing the initial ED laboratory and physiological variables [20]. Sequential organ failure assessment (SOFA) score was collected based on the worst variables of organ dysfunction recorded within 6 h of the ED visit [21, 22]. The calculation of PBS was determined by five components: mental status (alert, 0; disoriented, 1; stupor, 2; and coma, 4 points), body temperature (36.1°C–38.9°C, 0; 35.1°C–36.0°C or 39.0°C–39.9°C, 1; and ≤ 35°C or ≥ 40°C, 2 points), hypotension (drop in systolic blood pressure [SBP] > 30 mm·Hg and diastolic blood pressure > 20 mm·Hg or need of intravenous vasopressor agents or SBP < 90 mmHg, 2 points), mechanical ventilation (2 points), and cardiac arrest (4 points) [7]. The presence of a cardiac arrest event was defined as the occurrence of cardiac arrest on the day of or within the previous 48 h of the ED visit [13]. Each component was recorded using the worst point recorded within 24 h on the day of the ED visit. The summation of PBS ranged from 0 to 14, and the best mortality discriminative cutoff point was set as 4 point based on previous studies [7]. We then divided the nonbacteremic patients into low PBS (PBS < 4) and high PBS (PBS ≥ 4) groups for further analysis.

### 2.4. Outcome Measurement and Statistical Analyses

The primary outcome of this study was to examine the association between PBS and adverse outcomes in patients with nonbacteremic *K. pneumoniae* infections. We included the proportion of patients with septic shock (requiring vasoactive agents for hemodynamic support), ICU admission, renal replacement therapy, respiratory failure (requiring mechanical ventilation), and 30-day mortality as adverse outcomes. The secondary outcome was to compare the 30-day mortality discriminative ability of the PBS and other commonly used risk stratification scoring systems in patients with nonbacteremic *K. pneumoniae* infections. We included SIRS and qSOFA scores as they are easily obtained and widely used in the ED, and we also included the SOFA score as it is a well-validated scoring system for patients with critical illness [20–22]. The data were analyzed using the Statistical Package for the Social Sciences (SPSS, IL, USA), Version 25.0, and MedCalc Statistical Software Version 18.2.1 (MedCalc Software bv, Ostend, Belgium). Continuous variables with non-normal distribution were expressed as the median (interquartile range) or mean ± standard deviation with normal distribution. Between-group differences were compared using the two-sample *t*-test or Mann–Whitney test. Categorical variables were presented as percentages and analyzed using the Chi-square or Fisher's exact test (for *N* < 30). The 30-day mortality discriminative ability of the PBS and other scoring systems was examined using the area under the receiver operating characteristic (ROC) curves, and the DeLong method was used to compare the area under the curve (AUC) of these scoring systems. A two-tailed *p* < 0.05 was considered statistically significant.

## 3. Results

### 3.1. Study Population

**Figure 1:**
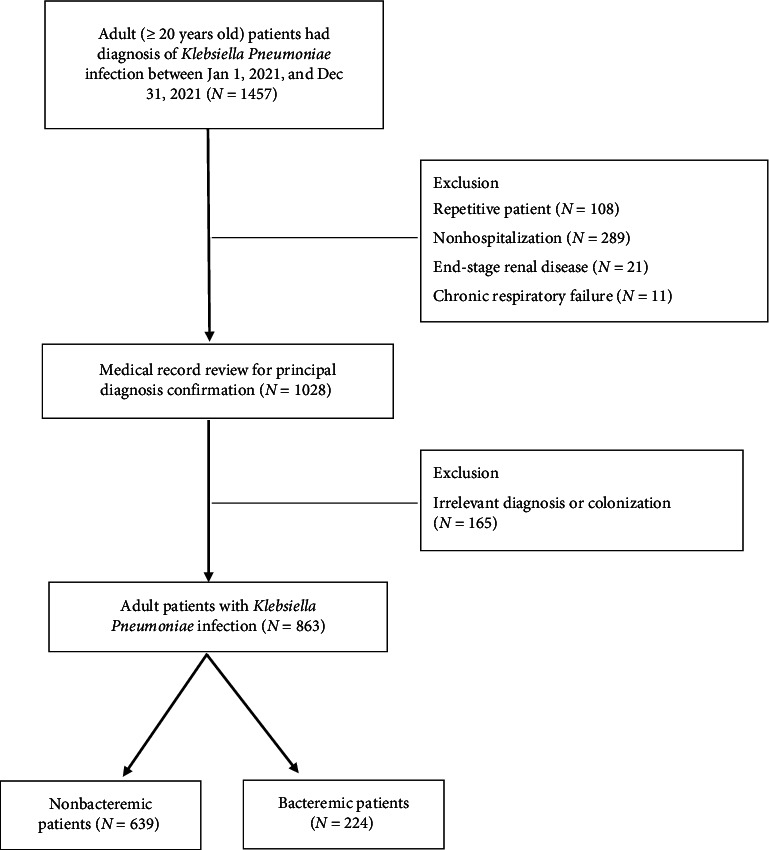
Flowchart of patient enrollment.

A total of 1457 adult patients with *K. pneumoniae* infection during the study period were included. After excluding repetitive patients (*N* = 108), those with end-stage renal disease (*N* = 21), chronic respiratory failure (*N* = 11), nonhospitalization (*N* = 289), and irrelevant diagnosis or colonization (*N* = 165), the remaining 863 patients with *K. pneumoniae* infection were divided into nonbacteremia (*N* = 639) and bacteremia (*N* = 224) groups for analyses (Figure 1).

### 3.2. Baseline Characteristics

The baseline characteristics were similar between nonbacteremic and bacteremic patients, except bacteremic patients had lower platelet count, higher lactate, C-reactive protein, and creatinine levels than nonbacteremic group (Supporting Table 1). As shown in Table 1, the mean age of nonbacteremic patients was 67.6 ± 16 years, and 53.8% (344/639) of the cohort was male. The most common comorbidity was hypertension (57.0%), followed by diabetes mellitus (46.3%) and chronic kidney disease (32.3%). Approximately half of the patients had urinary tract infections (44.3%), followed by skin/soft tissue (23.5%) and respiratory tract infections (22.1%). Patients were stratified into low PBS (< 4) and high PBS (≥ 4) groups for comparison. There were no significant differences in the proportion of comorbidities between the two groups, except that a higher proportion of patients with obstructive lung disease was observed in the high PBS group (11.8% vs. 6.0%, *p*=0.03). Regarding the laboratory results, patients in the high PBS group had significantly lower hemoglobin (10.4 [8.8–12.9] g/dL vs. 11.8 [10.0–13.5] g/dL, *p* < 0.01), higher leukocyte (12.1 [7.9–15.9] 10^9^/L vs. 10.2 [7.2–14.1] 10^9^/L, *p*=0.03), lactate (2.4 [1.5–4.8] mmol/L vs. 1.4 [1.0–2.3] mmol/L, *p* < 0.01), C-reactive protein (91.3 [41.6–167.5] mg/dL vs. 51.5 [14.9–123] mg/dL, *p* < 0.01), and creatinine levels (1.1 [0.7–2.1] mg/dL vs. 0.9 [0.7–1.4] mg/dL, *p* < 0.01) than those with low PBS. With respect to the infection source, patients in the high PBS group had a significantly higher proportion of respiratory tract infections (40.4% vs. 17.1%, *p* < 0.01) and a lower proportion of intra-abdominal (1.5% vs. 9.3%, *p* < 0.01) and skin/soft tissue (16.2% vs. 25.4%, *p*=0.02) sources of infection (Table 1). There were no significant differences in the proportion of resistant strains between the two groups.

### 3.3. Outcome Analyses

**Figure 2:**
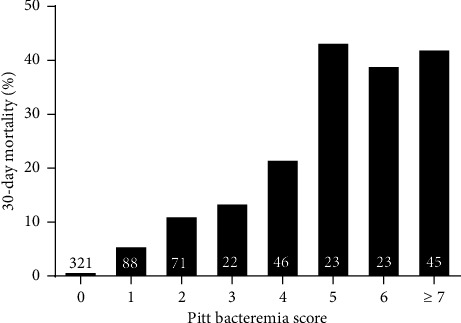
30-day mortality rate of patients with nonbacteremic *K*. *pneumoniae* infection in the Pitt bacteremia score group. Numbers in each bar represent the number of patients in the group.

The overall 30-day mortality rates of patients with *K. pneumoniae* infection were 13.2% (114/863) and 10.3% (66/639) in nonbacteremic patients. As shown in Table 2, compared with the low PBS group, patients in the high PBS group had a significantly higher risk of septic shock (77.9% vs. 4.8%, *p* < 0.01), ICU admission (71.3% vs. 8.2%, *p* < 0.01), renal replacement therapy (28.9% vs. 3.0%, *p* < 0.01), respiratory failure (71.3% vs. 2.4%, *p* < 0.01), and 30-day mortality (34.6% vs. 3.8%, *p* < 0.01). Moreover, the 30-day mortality risk of nonbacteremic patients showed a stepwise increasing trend as the PBS score increased (*p* < 0.01) (Figure 2), which showed a similar pattern to that in bacteremic patients (Supporting Figure 1).

### 3.4. Scoring Systems Comparison

**Figure 3:**
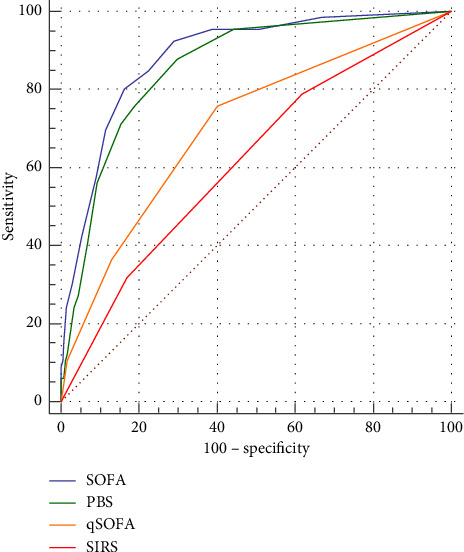
The 30-day mortality prediction ability of studied scoring systems for patients with nonbacteremic *K*. *pneumoniae* infection.

We further examined the 30-day mortality discriminative ability of PBS and other commonly used risk-stratification scoring systems in nonbacteremic patients with *K. pneumoniae* infection using ROC curves. As shown in Figure 3, the AUC of the studied scoring systems was as follows: SOFA 0.89 (95% confidence interval [CI] = 0.86–0.91), PBS 0.86 (95% CI = 0.83–0.88), qSOFA 0.71 (95% CI = 0.67–0.74), SIRS 0.62 (95% CI = 0.58–0.66). PBS revealed a significantly superior mortality prediction ability compared with qSOFA (*p* < 0.01) and SIRS (*p* < 0.01). Furthermore, the PBS score also had a better mortality prediction ability than qSOFA and SIRS in bacteremic patients with *K. pneumoniae* infection (Supporting Figure 2), indicating its prognostic significance in these patients.

## 4. Discussion

In this single-center, retrospective observational study, we investigated the prognostic value of PBS in patients with nonbacteremic *K. pneumoniae* infections. We revealed that high PBS was associated with renal dysfunction, acute inflammatory status, and tissue hypoperfusion in these patients and further correlated with the risk of adverse outcomes, including septic shock, ICU admission, renal replacement therapy, respiratory failure, and 30-day mortality. We also found that the PBS had an excellent mortality discriminative ability in these nonbacteremic patients, which was comparable to that of the SOFA score and superior to that of the SIRS and qSOFA scores. This is important because our findings broaden the application potential of PBS for risk stratification, not only in bacteremic patients but also in those with nonbacteremic infections.

*K. pneumoniae* infection is influenced by pathogen virulence, host intrinsic (age and immune status), and extrinsic factors [17]. Neonates, elderly people, and patients with immunosuppressive conditions such as diabetes, malignancy treated with chemotherapy, liver disease, chronic obstructive lung disease, renal insufficiency treated with dialysis, and poor nutritional status are deemed to be more susceptible to *K. pneumoniae* infection [1, 17]. The high rates of diabetes, chronic kidney disease, malignancy comorbidities in our patients, and the higher proportion of obstructive lung disease in the high PBS group were consistent with previous findings [1, 17] although the number of patients with chronic liver disease was unexpected low in our cohort, which requires future larger prospective studies for further validation [1].

The high proportion of urinary and respiratory tract infection sources in our patients was in line with previous studies [1, 2], whereas the high proportion of skin/soft tissue and intra-abdominal infections was unexpectedly low, which may be attributed to the distribution of their comorbidities [1]. *K. pneumoniae* strains are the second most common causative microorganisms of urinary tract infections, which were thought to be seeding from gastrointestinal tract [1, 23]. The growing prevalence of resistant strains seeded in the bladder makes these infections less susceptible to antimicrobial treatments, resulting in increased morbidity and prolonged hospitalization [1, 2]. Nevertheless, compared with respiratory tract or bloodstream infections, urinary tract infection is relatively minor, has little impact on the patient's general condition and leads to a low mortality risk [6]. The heterogeneous proportion of infection sources in our patients with nonbacteremic *K. pneumoniae* infections may explain their relatively low mortality rates [17].

Both the SIRS and qSOFA scores are widely used and easily accessible clinical evaluation parameters in patients with infectious diseases [24]. Compared with SIRS, the qSOFA score was developed for better accuracy in predicting mortality and ICU admission but had low sensitivity in detecting patients with infection-induced organ dysfunction [20, 25]. Whether SIRS or qSOFA scores have a better prognostic prediction ability in patients with infection remains debatable due to conflicting results [24, 25]. Importantly, there is a paucity of studies comparing them with other scoring systems for mortality discrimination in patients with a single causative pathogen, especially in those with nonbacteremic infections [26]. Our results provide evidence that both SIRS and qSOFA scores have only modest mortality discrimination ability in both bacteremic and nonbacteremic patients with *K. pneumoniae* infections, suggesting they may not be suitable as first-line screening tools for these patients.

The SOFA score was originally designed to assess the sequence of complications and monitor acute morbidity in patients with critical illness and has been extensively used and validated in various healthcare and environmental settings, including mortality risk prediction in patients with infectious diseases [21, 22, 27]. Each component of the SOFA score objectively describes the extent of vital organ dysfunction and thus has a strong correlation with organ failure and prognosis [27]. Therefore, it is not surprising that the SOFA score had the best mortality discrimination ability in our patients, including those in the bacteremic and nonbacteremic groups. Nonetheless, the intricacy of the calculation method, the fact that some patients may lack the required data because they are not routinely measured in the ED or ordinary ward, and concerns about the late identification of patients at risk may limit its use outside the critical care setting [28].

PBS is a highly significant predictor of mortality in patients with bacteremia [7]. The major advantage of the PBS is its simplicity, which is based solely on several patient-oriented parameters that can be calculated during an initial bedside physical examination, thus avoiding treatment and risk stratification delays in patients with infectious diseases [7]. Our study demonstrated that the PBS outperformed other commonly used quickly obtained risk scoring systems, including the SIRS and qSOFA scores, which further strengthened its prognostic value in these patients. Although rarely investigated previously, the significantly higher serum lactate, C-reactive protein, and creatinine levels in our high PBS group may indicate a correlation with organ dysfunction [7, 13]. In addition to the mortality risk, another important finding in our study was the clear association between PBS and other patient-centered adverse outcomes, including septic shock, ICU admission, respiratory failure, and renal replacement therapy. Furthermore, validation of the prognostic role of PBS in nonbacteremic patients could broaden its use in clinical scenarios since patients with nonbacteremic infections are estimated to be three to four times greater in number than those with bacteremia [13, 29].

We realize that there are some limitations in this study, including its retrospective single-institution design, which inevitably leads to recall and selection biases, and limit the applicability and generalizability of these results. Although we followed the criteria of site-specific *K. pneumoniae* infection, their differentiation from colonization may leads to selection bias [16, 17]. Other measures for sepsis management recommended by practice guidelines, including the type and amount of fluid administration, timing of vasopressor support, and implementation of source control measures, were not well calculated and controlled in this study [24]. Finally, some patients in the nonbacteremic group may have been misclassified because of inappropriate blood sample or culture methods [13].

## 5. Conclusions

In patients with nonbacteremic *K. pneumoniae* infection, high PBS was correlated with renal dysfunction, acute inflammatory status, and tissue hypoperfusion and further associated with septic shock, ICU admission, renal replacement therapy, respiratory failure, and 30-day mortality risk. The PBS outperformed commonly used risk stratification scoring systems, including the SIRS and qSOFA scores, and was comparable to the SOFA score in 30-day mortality discriminative ability in these patients. Physicians may utilize this simple scoring system for rapid assessment and prompt treatment of patients with infectious diseases.

## Figures and Tables

**Table 1:** Baseline characteristics of patients with nonbacteremic *Klebsiella pneumoniae* infection (*N* = 639).

Variables	All (*n* = 639)	Low PBS (PBS < 4) (*n* = 503)	High PBS (PBS ≥ 4) (*n* = 136)	*p* value
Age, y, mean ± SD	67.6 ± 16	67.1 ± 16.3	69.4 ± 14.8	0.13
Male, *n* (%)	344 (53.8)	272 (54.1)	72 (52.9)	0.82
Comorbidities, *n* (%)				
Diabetes mellitus	296 (46.3)	234 (46.5)	62 (45.6)	0.85
Hypertension	364 (57.0)	289 (57.5)	75 (55.1)	0.63
Chronic kidney disease	202 (32.3)	152 (30.6)	50 (38.8)	0.08
Chronic liver disease	29 (4.5)	22 (4.4)	7 (5.1)	0.65
Malignancy	152 (23.8)	113 (22.5)	39 (28.7)	0.13
Cerebrovascular accident	107 (16.7)	81 (16.1)	26 (19.1)	0.44
Obstructive lung disease	46 (7.2)	30 (6.0)	16 (11.8)	0.03^∗^
Laboratory results, median (IQR)				
Hemoglobin (g/dL)	11.5 (9.7–13.5)	11.8 (10.0–13.5)	10.4 (8.8–12.9)	< 0.01^∗^
Leukocyte (× 10^9^/L)	10.4 (7.3–14.4)	10.2 (7.2–14.1)	12.1 (7.9–15.9)	0.03^∗^
Platelet (× 10^9^/L)	224 (165–296)	228 (171–291)	211 (153–306)	0.38
Lactate (mmol/L)	1.6 (1.1–2.6)	1.4 (1.0–2.3)	2.4 (1.5–4.8)	< 0.01^∗^
Total bilirubin (mg/dL)	0.8 (0.5–1.8)	0.9 (0.5–2.0)	0.6 (0.4–1.3)	0.12
C-reactive protein (mg/dL)	60.2 (19.9–139)	51.5 (14.9–123)	91.3 (41.6–167.5)	< 0.01^∗^
Creatinine (mg/dL)	1.0 (0.7–1.5)	0.9 (0.7–1.4)	1.1 (0.7–2.1)	< 0.01^∗^
Infection source, *n* (%)				
Respiratory tract	141 (22.1)	86 (17.1)	55 (40.4)	< 0.01^∗^
Urinary tract	283 (44.3)	231 (45.9)	52 (38.2)	0.11
Intra-abdomen	49 (7.7)	47 (9.3)	2 (1.5)	< 0.01^∗^
Skin/soft tissue	150 (23.5)	128 (25.4)	22 (16.2)	0.02^∗^
Others	16 (2.5)	11 (2.2)	5 (3.7)	0.35
Resistant strains	96 (15.0)	72 (14.3)	24 (17.6)	0.15

*Note:* IQR, interquartile range.

Abbreviation: SD, standard deviation.

^∗^ *p* < 0.05.

**Table 2:** Outcome analysis of patients with nonbacteremic *Klebsiella pneumoniae* infection (*N* = 639).

Variables, *n* (%)	All (*n* = 639)	Low PBS (PBS < 4) (*n* = 503)	High PBS (PBS ≥ 4) (*n* = 136)	*p* value
Septic shock	130 (20.3)	24 (4.8)	106 (77.9)	< 0.01^∗^
Intensive care unit admission	138 (21.6)	41 (8.2)	97 (71.3)	< 0.01^∗^
Renal replacement therapy	54 (8.5)	15 (3.0)	39 (28.9)	< 0.01^∗^
Respiratory failure	109 (17.1)	12 (2.4)	97 (71.3)	< 0.01^∗^
30-day mortality	66 (10.3)	19 (3.8)	47 (34.6)	< 0.01^∗^

^∗^ *p* < 0.05.

## Data Availability

The data that support the findings of this study are available from the corresponding author upon reasonable request.

## Nomenclature

PBS: Pitt bacteremia score
CI: confidence interval
ED: emergency department
ICU: intensive care unit
eGFR: estimated glomerular filtration rate
SIRS: systemic inflammatory response syndrome
qSOFA: quick sequential organ failure assessment
SOFA: sequential organ failure assessment
SBP: systolic blood pressure
ROC: receiver operating characteristic
AUC: area under the curve

## Supporting Information

Supporting Information 1 Supporting Figure 1: The 30-day mortality rate of patients with bacteremic *K*. *pneumoniae* infection in the Pitt bacteremia score group. Numbers in each bar represent the number of patients in the group.

Supporting Information 2 Supporting Figure 2: The 30-day mortality prediction ability of studied scoring systems for patients with bacteremic *K*. *pneumoniae* infection.

Supporting Information 3 Supporting Table 1: Baseline characteristics of patients with *K. pneumoniae* infection (*N* = 863).
