# Supplementary material for: The Prognostic Role of Pitt Bacteremia Score in Patients With Nonbacteremic Klebsiella pneumoniae Infections
Source: Can J Infect Dis Med Microbiol. 2025 Jul 15;2025:6780766. doi: 10.1155/cjid/6780766 (PMC12283204; doi:10.1155/cjid/6780766)
Supplement: Supporting Information 1 — Supporting Figure 1: The 30-day mortality rate of patients with bacteremic K. pneumoniae infection in the Pitt bacteremia score group. Numbers in each bar represent the number of patients in the group. [file 6780766.f1.pdf]

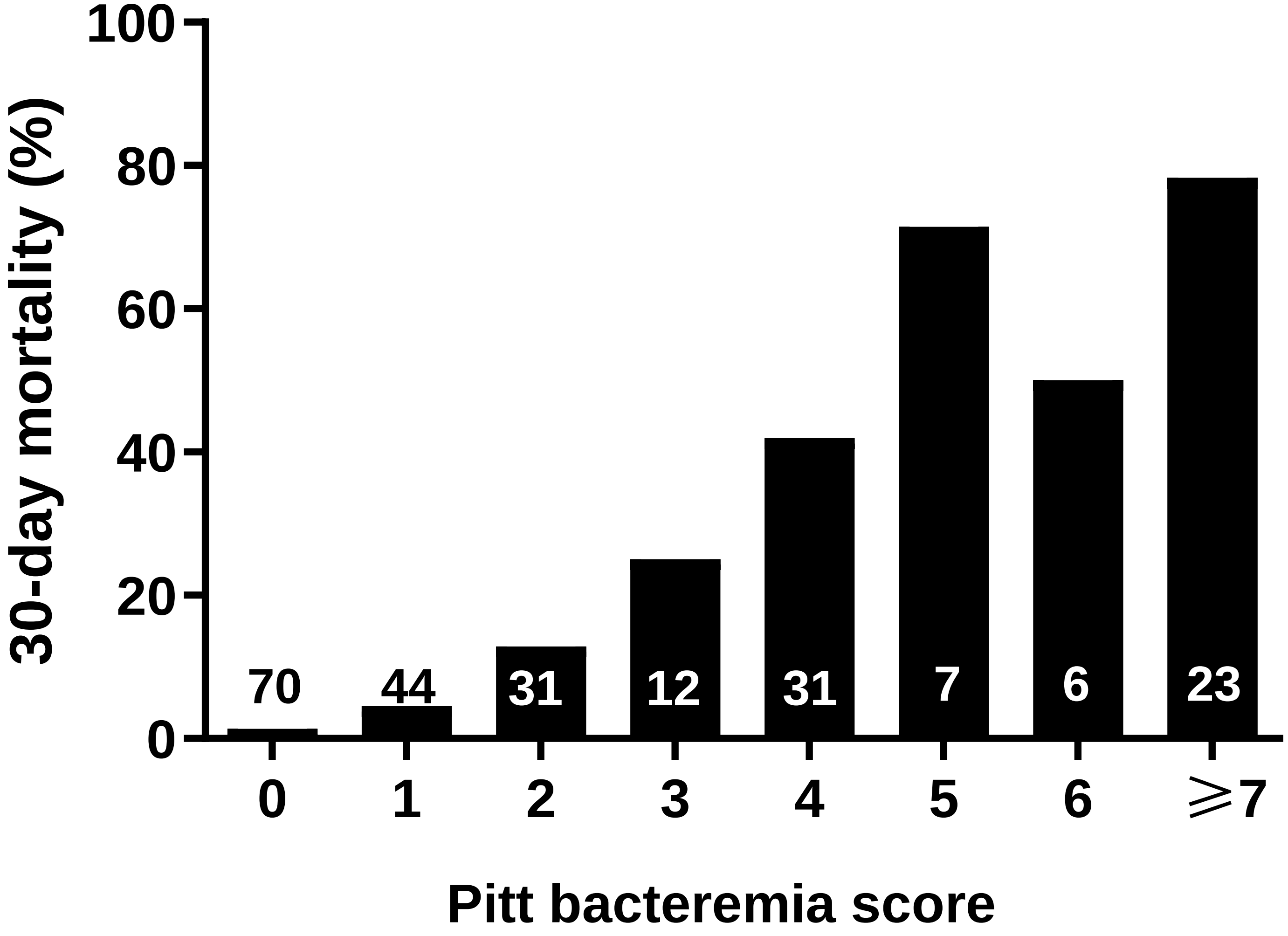

Supplementary Figure1. 30-day mortality rate of patients with bacteremic *k.pneumoniae* infection in the Pitt bacteremia score group.
Numbers in each bar represent the number of patients in the group
