# Supplementary material for: The Prognostic Role of Pitt Bacteremia Score in Patients With Nonbacteremic Klebsiella pneumoniae Infections
Source: Can J Infect Dis Med Microbiol. 2025 Jul 15;2025:6780766. doi: 10.1155/cjid/6780766 (PMC12283204; doi:10.1155/cjid/6780766)
Supplement: Supporting Information 2 — Supporting Figure 2: The 30-day mortality prediction ability of studied scoring systems for patients with bacteremic K. pneumoniae infection. [file 6780766.f2.pdf]

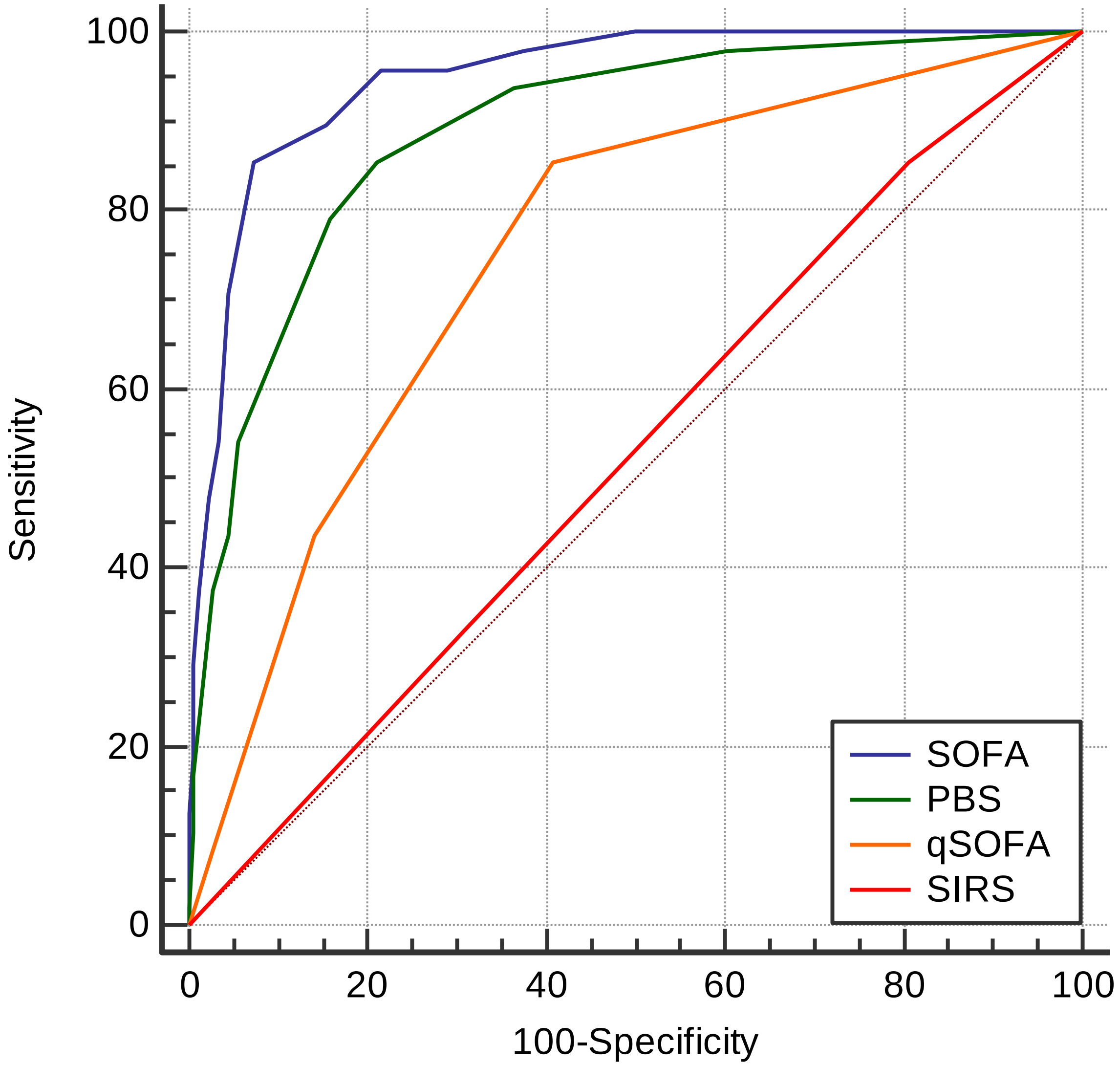

Supplementary Figure2. The 30-day mortality prediction ability of studied scoring systems for patients with bacteremic *k.pneumoniae* infection
