# Supplementary material for: The Prognostic Role of Pitt Bacteremia Score in Patients With Nonbacteremic Klebsiella pneumoniae Infections
Source: Can J Infect Dis Med Microbiol. 2025 Jul 15;2025:6780766. doi: 10.1155/cjid/6780766 (PMC12283204; doi:10.1155/cjid/6780766)
Supplement: Supporting Information 3 — Supporting Table 1: Baseline characteristics of patients with K. pneumoniae infection (N = 863). [file 6780766.f3.docx]

Supplementary Table 1. Baseline characteristics of patients with *K. pneumoniae* infection (N= 863)

| **Variables** | All  (n=863) | Non-bacteremia Bacteremia  (n=639) (n=224) | | *p* value |
| --- | --- | --- | --- | --- |
| Age, y, mean±SD | 67.2±15.7 | 67.6±16 | 66.0±15 | 0.16 |
| Male, n (%) | 460 (53.3) | 344 (53.9) | 116 (51.8) | 0.62 |
| Comorbidities, n (%)  Diabetes Mellitus  Hypertension  Chronic kidney disease  Chronic liver disease  Malignancy  Cerebrovascular accident  Obstructive lung disease  Laboratory results, median (IQR) | 403 (46.7)  494 (57.2)  287 (33.9)  44 (5.1)  213 (24.7)  143 (16.6)  61 (7.1) | 296 (46.3)  364 (57.0)  202 (32.3)  29 (4.5)  152 (23.8)  107 (16.7)  46 (7.2) | 107 (47.8)  130 (58.0)  85 (38.5)  15 (6.7)  61 (27.2)  36 (16.1)  15 (6.7) | 0.71  0.78  0.10  0.08  0.31  0.82  0.88 |
| Hemoglobin, g/dL | 11.6 (9.7-13.5) | 11.5 (9.7-13.5) | 11.8 (9.8-13.9) | 0.20 |
| Leukocyte, ×10^9^/L | 10.4 (7.3-14.8) | 10.4 (7.3-14.4) | 10.8 (7.3-16.2) | 0.60 |
| Platelet, ×10^9^/L | 212 (151-286) | 224 (165-296) | 164 (115-250) | <0.01* |
| Lactate, mmol/L  Total bilirubin, mg/dL | 1.8 (1.2-3.2)  1.0 (0.5-1.8) | 1.6 (1.1-2.6)  0.8 (0.5-1.8) | 2.5 (1.6-5.2)  1.1 (0.7-2.0) | <0.01*  0.10 |
| C-reactive protein, mg/dL  Creatinine, mg/dL | 69.1 (21.4-158)  1.0 (0.7-1.6) | 60.2 (19.9-139)  1.0 (0.7-1.5) | 102.2(30.6-202.8)  1.2 (0.8-1.9) | <0.01*  <0.01* |
|  |  |  |  |  |

**P*<0.05. SD: standard deviation. IQR: interquartile range.
